# Supplementary figures and images for: Repetitive mild traumatic brain injury affects inflammation and excitotoxic mRNA expression at acute and chronic time-points
Source: PLoS One. 2021 May 7;16(5):e0251315. doi: 10.1371/journal.pone.0251315 (PMC8104440; doi:10.1371/journal.pone.0251315)

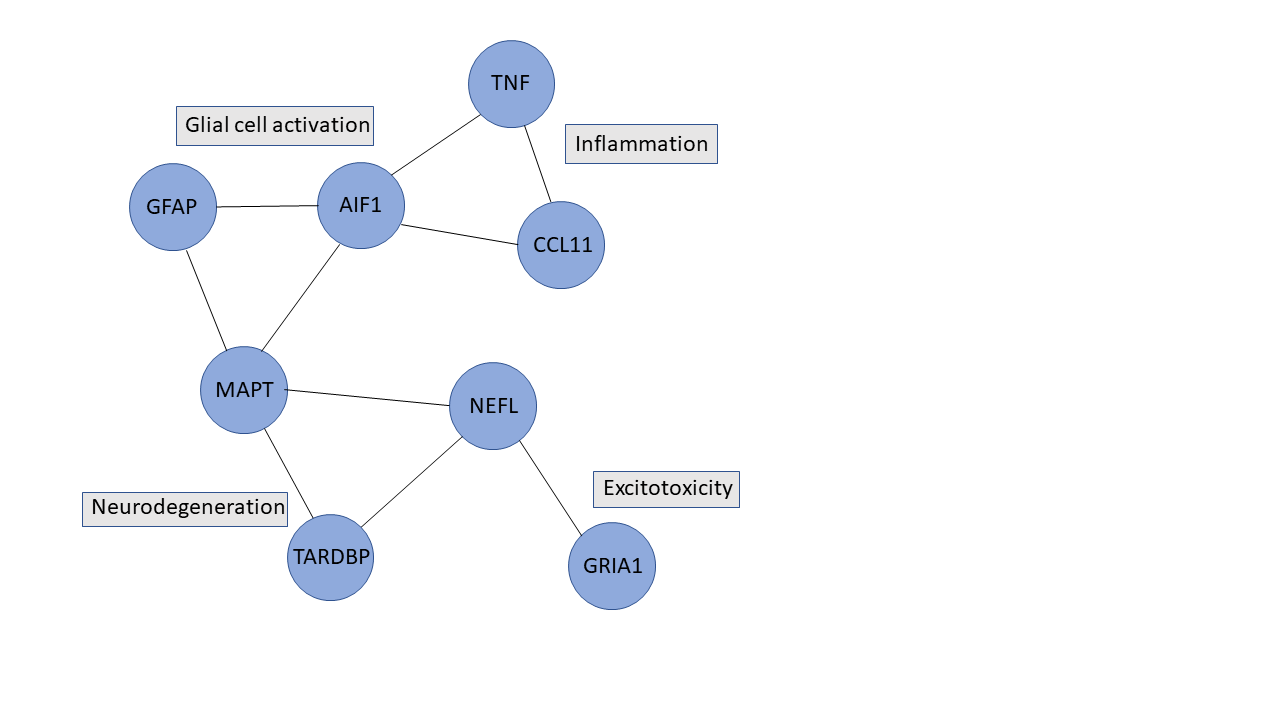

Supplement: S1 Fig — Edges have been defined based on co-expression, association in curated databases, or co-mentioned in publications. (TIF) [file pone.0251315.s001.tif]
